# Preface to special topic on lattice-based cryptography

**DOI:** 10.1093/nsr/nwab154

**Published:** 2021-09-09

**Authors:** Yu Yu

**Affiliations:** The Department of Computer Science and Engineering, Shanghai Jiao Tong University, China; Shanghai Qi Zhi Institute, China; Shanghai Key Laboratory of Privacy-Preserving Computation, China

Classical cryptography has been around for a long time in the documented human history, but most classical ciphers were broken and even solved by hand. Shannon introduced the notion of perfect secrecy that formally defines confidentiality in the information-theoretic sense, which is only possible in the restricted scenarios where the message is no longer than the encryption key. The invention of public-key cryptography (the Diffie-Hellman key exchange protocol in 1976 and the RSA crypto-system in 1977) marks the birth of modern cryptography, allowing parties to exchange messages securely without sharing any secrets in advance. Furthermore, it provides computational security based on the conjectured hardness of mathematical problems such as factorization and the discrete logarithm. Public-key cryptography has found numerous applications in the Internet, financial and banking industry, and blockchains, and it plays a crucial role in protecting information security and asset safety. Unfortunately, in the 1990s, Shor proposed efficient quantum algorithms that solve number-theoretic problems, including factorization and discrete logarithms in polynomial time. Once a quantum computer of a particular scale becomes a reality, it will cause a devastating blow to the existing public-key infrastructure. To deal with such a ‘quantum crisis’, academia and industry are looking into the design, analysis and standardization of cryptographic algorithms that can resist quantum computers referred to as post-quantum cryptography (PQC). The National Institute of Standards and Technology (NIST) has been soliciting proposals for the post-quantum public-key algorithms since 2016. More recently, the Chinese Association for Cryptologic Research (CACR) held a competition on designing cryptographic algorithms whose public-key cryptography track focused on post-quantum cryptographic algorithms. Lattice-based cryptography is considered by most to be the mainstream technical route of post-quantum cryptography, which is reflected in the number of proposals (and their percentage of the total) received in the NIST PQC process.

To reflect the status quo of post-quantum cryptography, we invite leading experts in this area to contribute three technical perspectives that aim to help readers understand the algorithms, the underlying basic techniques and different technical routes to achieve quantum resistance.

The first perspective, presented by Lu and Zhang, introduces public-key cryptographic algorithms whose quantum security is reducible from the conjectured quantum hardness of lattice problems. In particular, they mainly focus on public-key encryption (PKE) and the key encapsulation mechanism (KEM), which are essential building blocks for securing the confidentiality of communication without pre-shared secrets. Both types of crypto-systems are solicited by the NIST PQC standardization and the CACR algorithm design competition. This perspective gives a comprehensive survey on practical lattice-based PKEs/KEMs, and their best-known quantum and classical attacks.

Another important post-quantum crypto-system is digital signature, which ensures that three goals of information security are met other than confidentiality, namely, integrity, authentication and non-repudiation. The second perspective is on lattice-based signature by Lyubashevsky. In this perspective, he surveys different techniques in building lattice-based post-quantum crypto-systems, discusses the challenges in overcoming performance issues and gives us state-of-the-art digital signature schemes.

In addition to ensuring the ‘static’ security of information in storage and transmission, advanced cryptographic algorithms and protocols can guarantee information security during the computation process (possibly among multiple parties), referred to as privacy-preserving computation. Cryptographic techniques involved in privacy-preserving computation include secure multi-party computation, zero-knowledge proof and fully homomorphic encryption. There is a pressing need to migrate them to the post-quantum era. The third perspective, by Yu and Xie, presents practical instantiations of these algorithms and discusses possible ways to migrate them to their quantum-resistant counterparts.

To summarize, post-quantum cryptography has received widespread attention and made significant progress in recent years. Some post-quantum cryptographic algorithms, such as the lattice-based candidate, also have other advantages (e.g., computational efficiency and full homomorphism) over their classical counterparts. Lattice-based cryptography is an emerging field with high theoretical value and wide application, and we encourage young researchers to enter and explore this new and exciting field.

